# Do Guidelines Influence Emergency Department Staff Behaviours and Improve Patient Outcomes? Evaluation of a Multifaceted Intervention for the Implementation of Local Acute Exacerbations of Chronic Obstructive Pulmonary Disease Guidelines

**DOI:** 10.7759/cureus.3588

**Published:** 2018-11-13

**Authors:** Felix Zhou, Kavish Chandra, Dylan Sohi, Caitlin Robertson, Jacqueline Fraser, Josh Scoville, Natasha DeSousa, Chris Vaillancourt, Paul R Atkinson

**Affiliations:** 1 Emergency Medicine, Memorial University of Newfoundland, Saint John, CAN; 2 Emergency Medicine, Dalhousie University, Saint John, CAN; 3 Emergency Medicine, Saint John Regional Hospital, Saint John, CAN

**Keywords:** copd, emergency medicine, guideline adherence, guideline implementation, guidelines, clinical practice guidelines, chronic obstructive pulmonary disease, emergency department

## Abstract

Introduction

Published national guidelines on chronic obstructive pulmonary disease (COPD) highlight the importance of oxygen therapy, bronchodilators, corticosteroids, and appropriate antibiotics during acute exacerbations of COPD (AECOPD). We wished to assess how the implementation of local COPD guidelines affects emergency department (ED) staff awareness, knowledge, the use of such guidelines, and patient outcomes, including treatment failure and rates of return to the ED.

Methods

This study was conducted at a tertiary hospital ED. Local COPD guidelines were developed by a quality improvement group. Guidelines were posted in the department, and educational sessions were provided for staff. We conducted a retrospective chart review and looked at 1849 patient visits before and after COPD guideline implementation. All visits with a diagnosis of COPD or AECOPD were included in the study (for a total of 130 patient visits), and data were collected using a standardized abstraction tool. For non-admitted patients, we recorded 30-day return rates and treatment failures occurring within 30 days of presenting to the ED. Pre- and post-implementation outcomes were analyzed with Fisher’s exact tests. We also assessed ED staff awareness, knowledge, and use of COPD guidelines through surveys given out before implementation, and both one and 10 months after the implementation. We calculated proportions and 95% confidence intervals (CI) for our surveys. Pre- and post-implementation survey responses were compared with Fisher's exact tests.

Results

For ED physicians, the survey response rate was 78%, 79%, and 58% at pre-implementation, one-month follow-up, and 10-month follow-up, respectively. Prior to implementation, 14.3% (95% CI 4.1%-35.5%) were aware and 0% (0%-18.2%) reported using guidelines. One month after implementation, 90.9% (71.0%-98.7%) were aware and 81.8% (60.9%-93.3%) reported using guidelines. At 10 months, 100% (76.1%-100%) were aware and 100% (82%-100%) reported using local guidelines. Similar trends were seen among nurses and respiratory therapists. To assess actual guideline use, 130 visits were evaluated (51 visits prior to implementation and 79 post-implementation). Prior to implementation, 57% (43%-70%) received bronchodilators, systemic steroids, and antibiotics appropriately. Following guideline implementation, 57% (46%-67%) received the respective treatments (p=1.0). For patient-related outcomes, 86 non-admission patient visits were evaluated (35 visits prior to implementation and 51 post-implementation). Prior to guideline implementation, 17% (8%-33%) failed their initial AECOPD treatment, compared to 10% (4%-21%) following guideline implementation (p=0.34). Prior to guideline implementation, 23% (12%-39%) returned to the ED within 30 days in the pre-implementation period while 14% (7%-26%) returned post-implementation (p=0.39).

Conclusion

Our introduction of local COPD guidelines was successful at increasing self-reported awareness, knowledge, and the use of best practice guidelines among ED staff. At the 10-month follow-up, increased awareness, knowledge, and use of COPD guidelines among ED staff was maintained. However, in practice, guideline adherence, treatment failure, and return rates did not improve significantly after the implementation of local guidelines.

## Introduction

Chronic obstructive pulmonary disease (COPD) is a respiratory disorder that is largely caused by smoking but also by exposure to occupational dust and pollution. COPD is often undiagnosed, so current statistics are likely underestimates of the true prevalence. Self-reporting methods indicate that 4.4 percent of Canadians aged 35 years or older have COPD. In 2003, 5366 men and 4503 women were recorded as having died of COPD, making COPD the fourth leading cause of death in Canada [1]. COPD is a progressive disease, with gradual loss of lung function over time, but patients may experience more rapid declines after exacerbations where symptoms deteriorate acutely. These exacerbations are significantly different from day-to-day symptoms and include increased breathlessness, sputum production, and sputum purulence [2]. The etiology of at least half of acute exacerbations of COPD (AECOPD) are infectious in nature, but other triggers include congestive heart failure, pulmonary embolism, or exposure to allergens and irritants [3]. In Canada, AECOPD accounts for over 1.5 million physician visits annually [4]. Individuals with COPD who experience frequent exacerbations (more than two per year) have greater declines in health-related quality of life and are at higher risk for death [5]. In 1998, the economic burden of COPD in Canada was $1.67 billion and was mostly from emergency visits and hospitalizations related to AECOPD [6]. Thus, AECOPD poses a significant risk to health and growing healthcare costs.

For patients with AECOPD presenting to emergency departments (EDs), treatment choices by physicians are vastly important for immediate recovery as well as the reduction of treatment failure and risk of future exacerbations. The 2008 Canadian Thoracic Society guidelines recommend that patients with AECOPD be treated with combined, increased doses of inhaled, short-acting beta-agonists and an anticholinergic to improve dyspnea and pulmonary function [3]. Moreover, in patients with moderate to severe AECOPD, oral or parental corticosteroids for seven to 14 days are recommended. The treatment of AECOPD with systemic corticosteroids has been shown to improve airflow limitations, decrease treatment failure rates, and decrease the risk of relapse [7]. In patients with more severe purulent sputum, antibiotics are beneficial. Relapse rates were significantly higher (32%) in patients with severe exacerbations who were not given antibiotics, compared to those given antibiotics (19%) [8]. In the United States, it has been shown that only 60 percent of COPD patients received the recommended exacerbation care [9]. Thus, the development of concise local COPD guidelines for the treatment of AECOPD may improve the consistency of therapy and patient outcomes and reduce healthcare costs.

The aim of this study is to assess how the implementation of local COPD guidelines affects ED staff awareness, knowledge, and use of such guidelines, and patient outcomes, including treatment failure and rates of return to the ED. We hypothesize that local guideline implementation will increase staff awareness, knowledge, and use of guidelines and that these improvements will lead to better patient outcomes.

## Materials and methods

Study design and setting

We conducted an ambispective comparative study of patients presenting to the Saint John Regional Hospital (SJRH) ED with AECOPD from December 2011 to March 2012 and December 2012 to March 2013. Because AECOPD rates vary significantly by season, we matched the pre-implementation and post-implementation study periods by month. The SJRH is a tertiary teaching hospital. Concise, local COPD guidelines were implemented between the two time periods, in November 2012. A multifaceted intervention was used, in which printed copies of the guidelines and order sets were circulated and posted in the department, and a series of educational sessions were provided for ED physicians, nurses, and respiratory therapists. These sessions included a literature review, rationale for guideline development, and review of the diagnosis and treatment of AECOPD.

Study population

A registry was obtained from the local hospital patient administration system that included any patient registered initially to the SJRH ED with Canadian Emergency Department Information System (CEDIS) complaints of shortness of breath, respiratory arrest, cough/congestion, hyperventilation, hemoptysis, stridor, wheezing, upper respiratory tract infection complaints, chest pain (non-cardiac), or general weakness. All patient charts with a diagnosis of chronic COPD or AECOPD were included in the study. Exclusion criteria for this study were similar to those in previous literature [8] and included anyone under the age of 40 (because the onset of COPD is later in life, usually over 40 years of age) and patients thought to have shortness of breath due to pneumonia or congestive heart failure.

Patient demographics were collected from charts and included a history of smoking, current smoking status, dependence on home oxygen, and comorbidities such as diabetes, hypertension, coronary artery disease, congestive heart failure, cardiovascular disease, cancer, renal insufficiency, and liver disease.

Outcome measures

**Figure 1:**
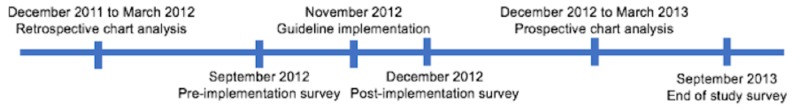
Timeline of our study from December 2011 to September 2013

We assessed ED staff awareness, knowledge and use of COPD guidelines using online and paper surveys as part of a continuous quality improvement initiative by the ED. The surveys are contained in Appendix 1. Surveys were given out before the implementation of COPD guidelines (September 2012), one month following implementation (December 2012), and 10 months following implementation (September 2013). See Figure 1 for an overview of the timeline of the study.

Patient outcomes were collected and included treatment failure and a recurrence of AECOPD. As in previous studies [10], treatment failure was defined as the intensification of drug treatment, readmission for COPD, intubation or death within 30 days of discharge. Because of the large variability in care for patients when they are transferred from the ED to inpatient units within the hospital, we only assessed outcomes in patients who were discharged home directly from the ED. For both patients discharged from the ED and admitted to hospital, we determined the percentage of patients who received proper treatment based on the guideline recommendations.

Initially, standard chart identifiers (name, date of birth, and unique personal number) were accessed. After data collection, all patients were given a study number, which was used subsequently for data analysis. The study was conducted in accordance with the International Conference on Harmonization for Good Clinical Practice and the appropriate regulatory requirements. Approval from the Horizon Research Ethics Board was obtained (Horizon Health Network File #2012-1803).

Statistical analyses

Descriptive statistics were calculated for all demographic variables, including a history of smoking, current smoking status, oxygen dependence, and comorbidities. We calculated proportions and 95% confidence intervals (CI) for our surveys, assessing ED staff awareness, knowledge, and use of guidelines. Pre- and post-implementation survey responses were compared with chi-square analyses. We compared patient outcomes (treatment failure, rate of return to ED) between pre- and post-implementation groups using chi-square analyses.

## Results

Baseline demographic characteristics

There were 1849 patients registered to the SJRH ED with CEDIS complaints of shortness of breath (SOB), respiratory arrest, cough/congestion, hyperventilation, hemoptysis, stridor, wheezing, upper respiratory tract infection complaints, chest pain (non-cardiac), or general weakness in our study period. Of these 1849 patients, 130 were diagnosed with AECOPD or COPD.

The demographic details of our study population are described in Table 1. There were 51 and 79 patients in our pre- and post-implementation groups, respectively.

**Table 1:** Baseline demographic characteristics of the study population.

Demographics	Pre-implementation	Post-implementation
Positive smoking history	40/51 (78%)	60/79 (76%)
Current smoker	26/51 (51%)	30/79 (38%)
Oxygen dependent	9/51 (18%)	10/79 (13%)
One comorbidity	7/51 (14%)	7/79 (9%)
Two comorbidities	6/51 (12%)	16/79 (20%)
Three or more comorbidities	38/51 (75%)	56/79 (71%)

Self-reported awareness, knowledge, and use of local COPD guidelines before and after implementation

**Figure 2:**
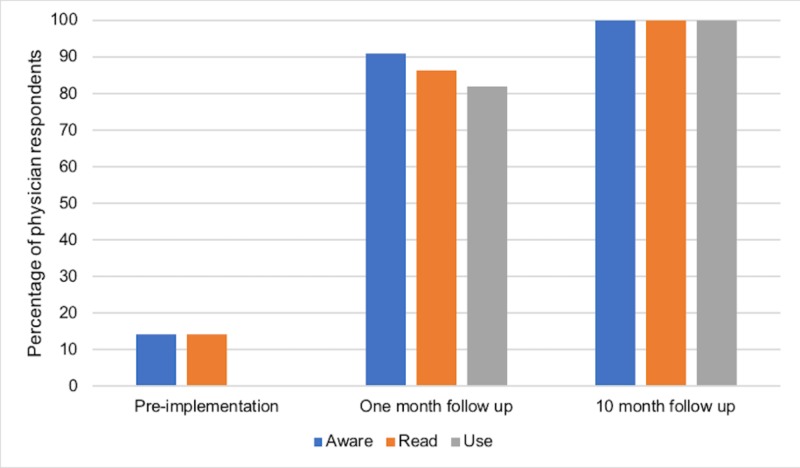
Guideline awareness, knowledge, and use among ED physicians before, one month after, and 10 months after implementation of local COPD guidelines ED: emergency department; COPD: chronic obstructive pulmonary disease

For ED physicians, the survey response rate was 78, 79, and 58% at pre-implementation, one-month follow-up, and 10-month follow-up, respectively. Prior to implementation, 3/21 physicians (14.3%; 95% CI 4.1%-35.5%) were aware and 0/21 (0%; 0%-13.5%) reported using the COPD guidelines (Figure 2). One month after implementation, 20/22 (91%; 71%-98.7%) were aware and 18/22 (82%; 60.9%-93.3%) reported using the COPD guidelines. This improvement in awareness and reported use of COPD guidelines from pre-implementation to one-month follow-up was statistically significant (p<0.0001). At 10 months, 15/15 (100%; 82%-100%) were aware and 15/15 (100%; 82%-100%) reported using local guidelines. This change from one month to 10 months post-implementation was not statistically significant.

**Figure 3:**
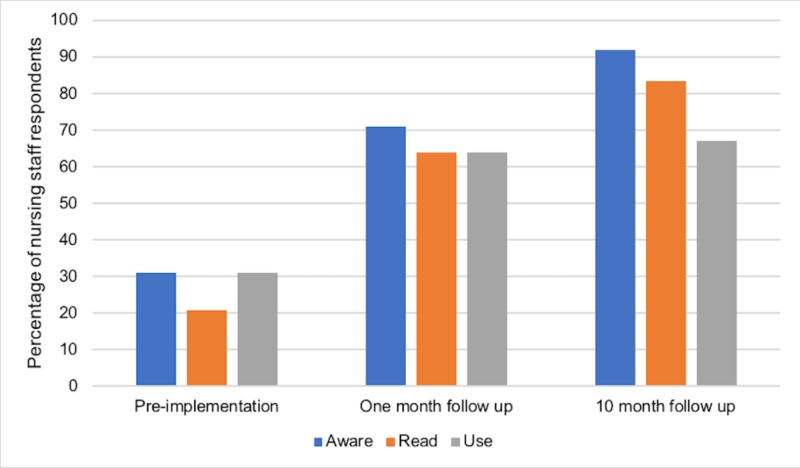
Guideline awareness, knowledge, and use among ED nurses before, one month after, and 10 months after implementation of local COPD guidelines ED: emergency department; COPD: chronic obstructive pulmonary disease

For ED nursing staff (NS), the response rate was 39, 19, and 16% at pre-implementation, one-month follow-up, and 10-month follow-up, respectively. Prior to implementation, 9/29 NS (31%; 17.1%-49.4%) were aware and 9/29 (31%; 17.1%-49.4%) reported using guidelines (Figure 3). One month after implementation, 10/14 NS (71%; 45%-88.7%) were aware and 9/14 (64%; 38.6%-83.8%) reported using guidelines. This improvement in awareness from pre-implementation to one-month follow-up was statistically significant (p<0.0001). At 10 months, 11/12 NS (92%; 62.5%-99.9%) were aware and 8/12 (67%; 38.8%-86.5%) reported using guidelines. This improvement in the reported use of guidelines from one month to 10 months post-implementation was statistically significant (p<0.001).

**Figure 4:**
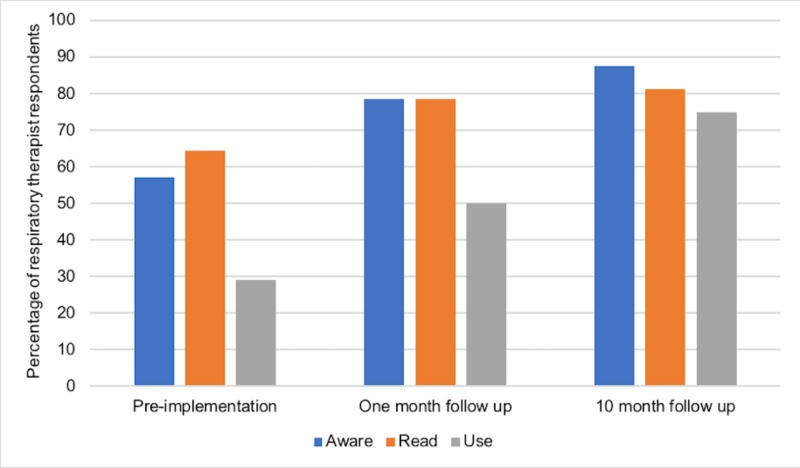
Guideline awareness, knowledge, and use among ED respiratory therapists before, one month after, and 10 months after implementation of local COPD guidelines ED: emergency department; COPD: chronic obstructive pulmonary disease

For respiratory therapists (RTs), the response rate was 24, 24, and 28% at pre-implementation, one-month follow-up, and 10-month follow-up, respectively. Prior to implementation, 8/14 RTs (57%; 32.6%-78.7%) were aware and 4/14 (29%; 11.3%-55.0%) reported using guidelines (Figure 4). One month after implementation, 11/14 RTs (78.6%; 51.7%-93.2%) were aware and 7/14 (50%; 26.8%-73.2%) reported using guidelines. At 10 months, 14/16 RTs (87.5%; 62.7%-97.8%) were aware and 12/16 (75%; 50.0%-90.3%) reported using guidelines. This improvement in the reported use of guidelines from one month to 10 months post-implementation was statistically significant (p<0.001).

Influence of local COPD guideline implementation on guideline adherence, treatment failure, and ED return rates

**Figure 5:**
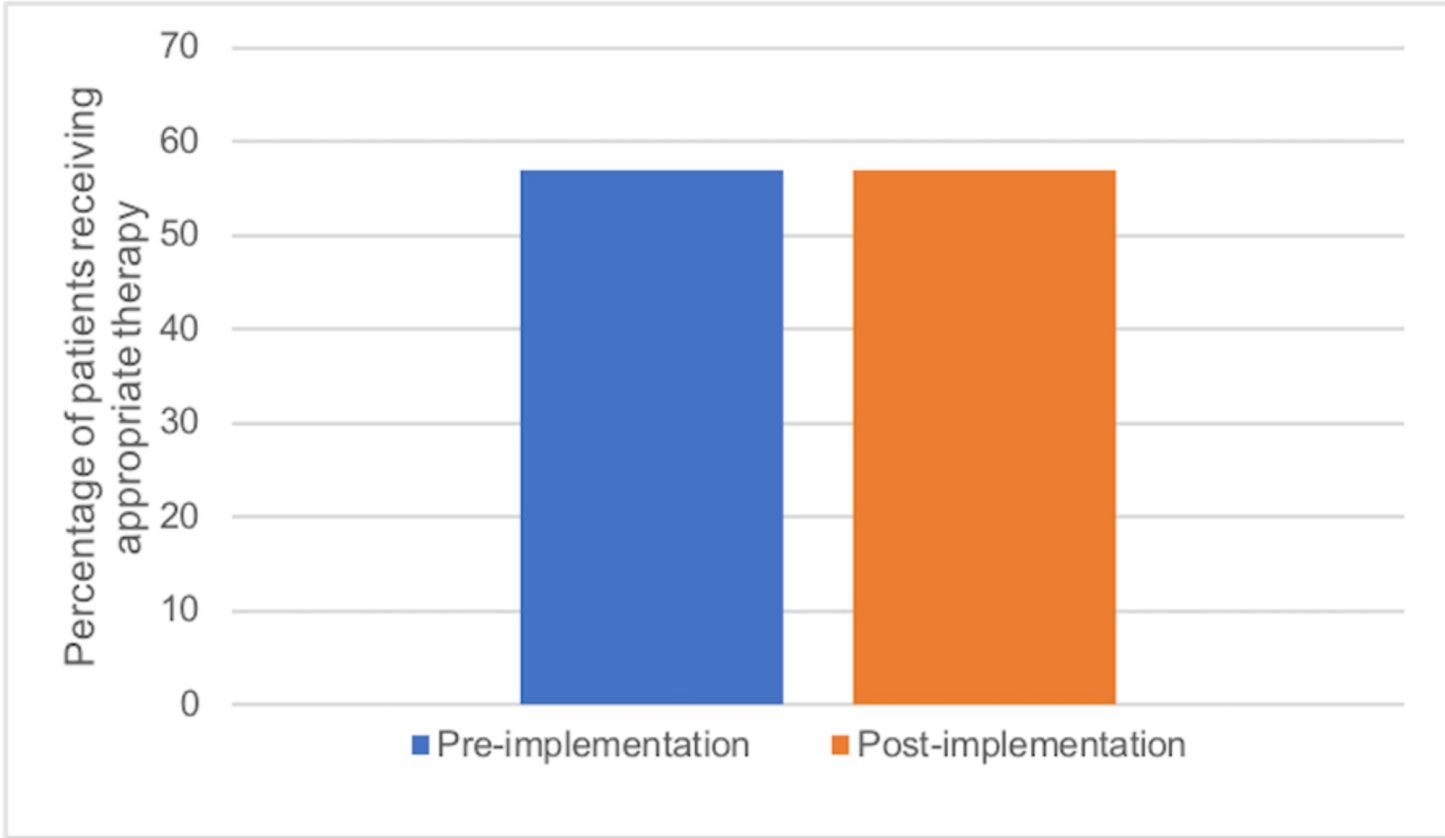
Percentage of patients receiving appropriate AECOPD therapy before and after implementation of local COPD guidelines COPD: chronic obstructive pulmonary disease; AECOPD: acute exacerbations of COPD

To determine the influence of local COPD guideline implementation on guideline adherence, we evaluated 130 visits, with 51 visits occurring prior to implementation and 79 visits post-implementation. Prior to implementation, 29/51 patients (57%; 43.3%-69.5%) received appropriate therapy with bronchodilators, systemic steroids, and antibiotics (Figure 5). Following guideline implementation, 45/79 patients (57%; 46%-67.3%) received the appropriate treatment (p=1.0; Figure 5).

**Figure 6:**
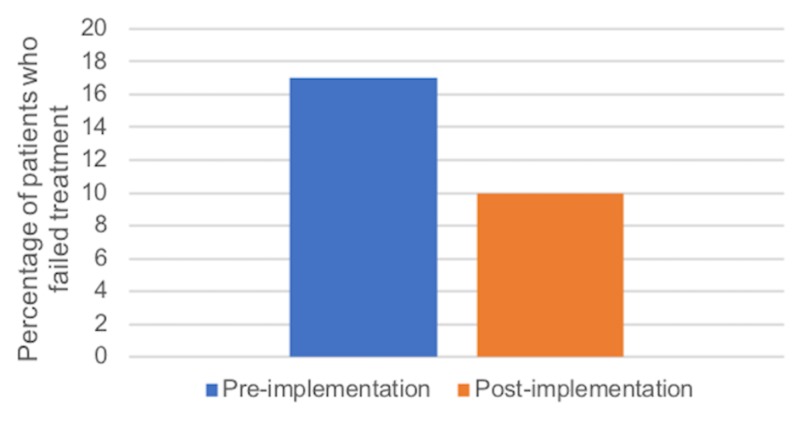
Percentage of patients who failed initial treatment before and after implementation of local COPD guidelines COPD: chronic obstructive pulmonary disease

**Figure 7:**
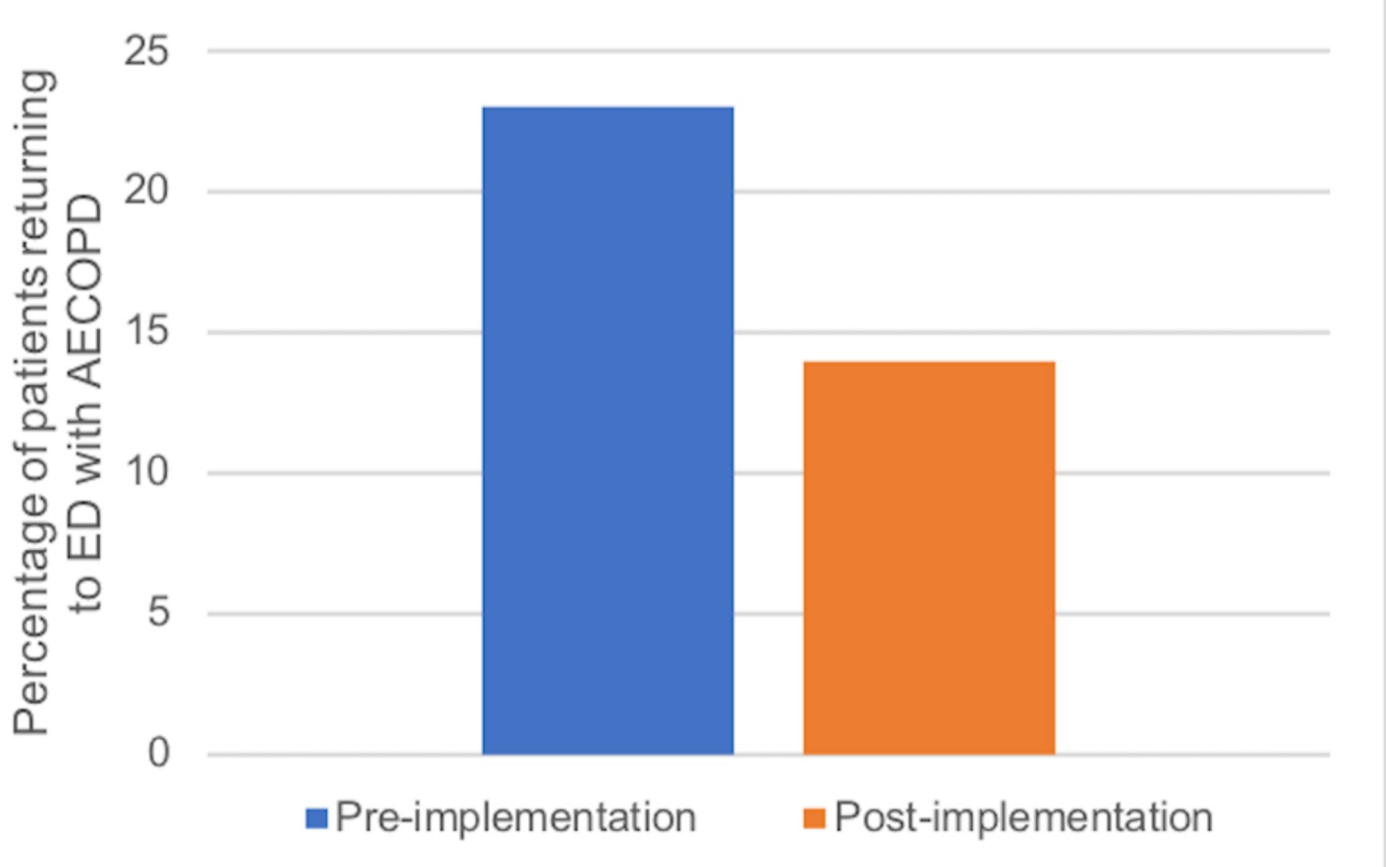
Percentage of patients returning to the ED for AECOPD within 30 days of discharge before and after implementation of local COPD guidelines ED: emergency department; COPD: chronic obstructive pulmonary disease; AECOPD: acute exacerbations of COPD

To determine the influence of local COPD guideline implementation on treatment failure and ED return rates, we evaluated 86 visits, with 35 visits occurring prior to implementation, and 51 visits post-implementation. Prior to implementation, 6/35 patients (17%; 7.7%-33.1%) failed their initial AECOPD treatment, compared to 5/51 patients (10%; 3.8%-21.4%) following implementation (Figure 6). There was no statistically significant difference in treatment failure between the pre- and post-implementation groups (p=0.34). Prior to implementation, 8/35 patients (23%; 11.8%-39.3%) returned to the ED within 30 days for an AECOPD-related complaint compared to 7/51 patients (14%; 6.5%-26%) following implementation (Figure 7). There was no statistically significant difference in ED return rates between the pre- and post-implementation groups (p=0.39).

## Discussion

The primary goal of our study was to determine whether the development of concise local COPD guidelines would improve ED staff awareness, knowledge, and use of such guidelines and ultimately improve patient outcomes. Our surveys showed that self-reported ED staff awareness, knowledge, and use of guidelines improved immediately following the implementation and that this benefit was retained 10 months after (Figure 2-4). However, our ambispective chart review revealed that guideline adherence, treatment failure, and return rates did not improve significantly after implementation (Figures 5-7). There are multiple factors why self-reported awareness, knowledge, and use of guidelines may have improved but not led to improved adherence upon chart review. It is likely easier to impact the awareness of information than it is to change actual behavior. Changing behavior is a complex process involving many steps in addition to the intervention itself, including a thorough needs assessment and an identification of barriers to change [11] There is a growing body of evidence that recognizes the importance of a more holistic approach to stimulating behavioral change, termed knowledge translation [11]. Knowledge translation can be defined as a dynamic and iterative process that closes the gaps from knowledge to practice [12]. While there are several frameworks for the practice of knowledge translation, in general, it includes the assessment of barriers to change, the creation of tailored interventions, evaluating outcomes, and sustaining knowledge use [12]. Knowledge translation targets a wide audience that includes not only physicians but also patients, consumers, and policy makers [11]. Our study evaluated the effectiveness of an educational intervention consisting of printed educational materials (PEMs) and brief educational sessions and was not an evaluation of a more comprehensive knowledge translation initiative.

There are a variety of different formats through which guidelines may be implemented, including PEMs, didactic lectures, audit and feedback, and workshops. The wide variation in methodology in the literature makes it difficult to draw conclusions regarding the effectiveness of these different modalities. Some studies evaluate single interventions while others evaluate multifaceted interventions, combining modalities such as PEMs with workshops. A recent systematic review concluded that multifaceted, interactive education and clinical reminder systems were effective while didactic education and passive dissemination strategies were ineffective [13]. A review of 45 studies found that PEMs alone (as compared to no intervention) only had a small beneficial effect on professional practice [14]. Our study supports the general trend in the literature, showing that passive educational interventions alone are not sufficient to change physician behaviors or improve patient outcomes.

Our study had several limitations. This was a single-center study, which may limit the generalizability of our findings. A larger sample size may have allowed us to uncover statistically significant improvements in patient outcomes. We collected baseline demographic characteristics (current smoking status, oxygen dependence, comorbidities, etc.) to gauge the distribution of these factors among the pre- and post-implementation groups. However, a larger sample size would have facilitated the development of logistic regression models that incorporate the baseline demographic characteristics as possible confounding factors in our evaluation of local guideline effectiveness.

Despite these limitations, our study demonstrated that a self-reported awareness of guidelines does not necessarily translate into a change in clinical practice and, ultimately, patient outcomes. While clinical practice guidelines may provide evidence-based recommendations to clinicians who are faced with the challenge of keeping up with rapidly evolving fields, it is important to evaluate the effectiveness of the implementation of these guidelines. There are a variety of ways that guidelines may be implemented, from less expensive methods, including PEMs, to more resource-intensive interventions, such as audit and feedback or workshops. While still a relatively new field, knowledge translation initiatives offer a more rigorous and holistic approach to facilitating behavioral change and may be more effective than traditional passive educational interventions.

## Conclusions

Our study showed that local COPD guideline implementation improved the self-reported awareness of guidelines but did not affect guideline adherence in practice or patient outcomes. This supports the general trend in the literature showing that passive educational interventions alone are not sufficient to change physician behaviors or improve patient outcomes. While still a relatively new field, knowledge translation initiatives offer a more rigorous and holistic approach to facilitating behavioral change and may be more effective than traditional passive educational interventions. Future studies should evaluate the effectiveness of knowledge translation initiatives in improving patient outcomes, in addition to physician behavior, and in analyzing the cost-effectiveness of these more resource-intensive interventions. In the meantime, those who are in charge of making decisions regarding the implementation of guidelines should consider the local burden of disease, current guideline adherence rates, and assess the effects of any interventions.

## Appendices

Appendix 1: survey of EM and nursing staff awareness, knowledge, and use of COPD guidelines

Name (first and last):

1)    Are you aware of national COPD guidelines?

2)    Have you read national COPD guidelines?

3)    Do you use national COPD guidelines?

4)    Are you aware of local COPD guidelines?

5)    Have you read local COPD guidelines?

6)    Do you use local COPD guidelines?

Please note that respondents will answer yes or no to questions.
